# Assisting primary care teams and patients in a culturally diverse periphery: impact on medical students’ future career choices

**DOI:** 10.1186/s12909-024-05272-x

**Published:** 2024-03-14

**Authors:** Nosaiba Rayan-Gharra, Lilach Malatskey, Marganit Ofir-Gutler, Rizan Sakhnini, Awni Yousef, Mohammad Khatib, Karl Skorecki, Sivan Spitzer

**Affiliations:** 1 Azrieli Faculty of Medicine, Bar-Ilan University, Zefat, Israel; 2 The Cheryl Spencer Department of Nursing, University of Haifa, Haifa, Israel; 3 Maccabi Health Services, Northern District, Haifa, Israel; 4 Clalit Health Services, Northern District, Nazareth, Israel; 5 The Galilee Society - The Arab National Society for Health Research and Services, Shefa-Amr, Israel; 6 Zefat Academic College, Zefat, Israel

**Keywords:** Medical students, Medical education, Chronic disease, Social determinants of health, Primary care

## Abstract

**Background:**

Medical students can assist in reducing healthcare disparities and promote health equity by engaging with rural communities and gaining insights into their unique healthcare needs. A two-arm student-delivered program was designed and implemented during COVID-19 in a social-geographic peripheral area to assist clinics with complex chronic and/or socially disadvantaged patients and improve preventive behavior in townships through home visits delivering community kits.

**Methods:**

We conducted a pre-post design study which included weekly structured medical student reports and monthly structured telephone interviews with clinic directors and municipal partners. Students completed pre-post program survey on their knowledge, skills, and capabilities to address chronic patients from diverse cultural backgrounds (*n* = 73). The Wilcoxon-Signed-Rank test for related samples was used to determine differences.

**Results:**

Following the program, the knowledge and awareness levels of students about working in the community (*P* < 0.001) and their knowledge of common chronic diseases were significantly improved (Mean Difference (MD) = 0.31; *p* < 0.001). The program significantly increased students’ interest to integrate into community care alongside a hospital (*P* = 0.012). Thematic analysis of student reports revealed improved insight into the role of primary care. Clinic directors (90%) were highly satisfied and reported that students became an integral part of the clinics’ teams.

**Conclusions:**

Integrating medical students into the community through primary-care clinics and home visits in diverse communities, exposed students to the interwoven effect of clinical and social determinants on health and improve their knowledge of common chronic diseases. Participation in the program encouraged students to consider a career in community care.

## Background

Social determinants coupled with complex health needs deepen disparities among those already disadvantaged [1]. The role of medical schools, and more specifically that of medical students, in reducing health inequities has been widely debated.

Medical students can assist in reducing healthcare disparities and promoting health equity by engaging with rural communities and gaining insights into their unique healthcare needs [2]. Studies found that medical students who took part in a student-run faculty-collaborative clinic helping low income, low health literacy, immigrant, and refugee populations, reduced unnecessary emergency department utilization [3], reinforced positive health behaviors, and improved health information-seeking habits [4]. Engaging with rural or social peripheral communities has been found to enhance medical students’ community engagement skills [5], impact their career choices to choosing primary care or family medicine [6], encourage them to stay and work in peripheral or rural areas [7, 8], and as such improve healthcare access and health outcomes for underserved populations [2].

Covid-19 served as an opportunity to involve students in interventions aimed at reducing the widening inequities brought on by the pandemic. Medical schools’ differed in their preferred strategies [2]. Some schools chose not to allow students to interact with patients while others graduated students early so that they could join frontline staff [9–12]. This debate deepened for medical schools located in social-geographic peripheries where health inequities were exacerbated [13]. Outreach programs conducted by family medicine residents as well as medical students in disadvantaged communities during COVID-19 can be found [14–16], However, most focused on the impact interventions had on patient outcomes, and the impact such interventions had on medical students future professional preferences is limited.

A unique participatory community-based intervention to reduce inequities in a social-geographic periphery:

The Azrieli Faculty of Medicine (aka Faculty) was established in the Galilee region, Israel’s northern periphery, laying the foundation for a unique participatory community-based intervention to reduce inequities in a social-geographic periphery. The Galilee region’s societal composition of diverse minority groups, low socioeconomic status, religiosity, low health literacy, and markedly high prevalence of chronic morbidity, overstretched and challenged healthcare providers in their ability to provide care and support. These conditions presaging an impending crisis of backlog of chronic non-communicable non-COVID-19 disease [17]. To address these challenges, the Faculty convened a roundtable comprised of representatives from its six affiliated hospitals, affiliated primary care clinics operated by Israel’s two largest health maintenance organizations (HMOs), as well as local NGOs and municipalities. This enabled a broad regional overview of needs and resources at each of these otherwise siloed sites.

In April 2021 a two-arm regional student-delivered psycho-social-health program was designed and implemented: (1) Students conducted a support service to assist primary care teams in chronic care patient management was set up. Our partner HMOs identified clinics with a particularly high percentage of complex chronic conditions and/or socially disadvantaged patients requiring support; (2) To improve adherence to COVID-19 preventative behavior in Arab townships with high morbidity identified by our partner NGO. Students conducted home visits to elderly residents, disseminating community kits that included specially prepared plain language pamphlets in Arabic, thermometers, and masks. Given the unique opportunity to shed light on the impact of the program on medical students, we assessed the program’s contribution to clinical-social knowledge as well as future occupational intentions.

## Methods

### Participants, context, and program structure

Medical students from the clinical years who enrolled in the program were recruited to assist primary care clinics’ teams, and students who enrolled from the pre-clinical years focused on improving COVID-19 preventive behavior adherence in Arab townships. Students received a stipend from the faculty of medicine for their participation in the program.

Students in their clinical years assisted family physicians and nurses in reaching out to chronic patients through phone calls and home visits during lockdowns. They also took part in daily clinic activities to reduce major COVID-19 backlog due to COVID-19 such as monitoring patients’ quality of care including recommended routine screening (e.g. mammography or fecal occult blood testing for detection of colon cancer) as well as monitoring chronic patient measures (e.g. hypertension blood pressure or hemoglobin A1C). They helped enroll patients in health promotion workshops to stop smoking and promote diet and physical activity changes. They also participated in clinic staff rotations to monitor patients’ temperature upon entry to the clinic and to monitor COVID-19 patients who were at home.

Prior to beginning their placements, students participated in a preparatory meeting with regional and sub-regional directors of the HMOs as well as Faculty leaders to better understand the needs and expectations of the HMO and region in which they would be working. Maccabi Health Care provided for students five additional online training workshops with leading specialists. Workshops included skill-based training on chronic patients’ monitoring and quality indicators, working with diabetic patients and monitoring via telephone risks of vascular complications, medication treatment of dyslipidemia, and renal failure.

In partnership with the Galilee Society, the pre-clinical students launched an intervention to increase awareness and adherence to COVID-19 preventative behavior in Arab townships across the Galilee. The intervention included home visits to elderly residents, passing out community kits that include disposable masks, a thermometer and information leaflets that were created in plain language in Arabic on COVID-19 and on chronic illness management during COVID-19.

The Galilee Society identified 16 Arab towns for the intervention and eleven pre-clinical students were recruited to assist their social services departments. Even though these towns initially confirmed their participation, often they were delayed in launching the program and, did not always have sufficient manpower to accompany the students. Ultimately, four municipalities decided in the end not to implement the program. The program began in August, with the rise in COVID-19 morbidity in the Arab population. Over 800 home visits were planned in Sakhnin and Mag’d Al Krum. Students received online zoom training from Dr. Muhamed Khatib of the Galilee Society and Dr. Sivan Spitzer to better understand the COVID-19 situation in the Arab community, the difference in morbidity and population behavior between the first and second wave, and cultural competency training for conducting home visits in the Arab sector. Students prepared the community kits to be passed out in these communities.

### Data collection

#### Quantitative data

Students completed pre and post program questionnaires on their knowledge, skills, and capabilities to address chronic patients from diverse cultural backgrounds. Eleven questions adapted from Dent et al. (2010) were identified as relevant to this program: attitude assessment (two items), intent to practice (4 items), and knowledge assessment (5 items). Responses were measured using a 4-point Likert scale ranging from 1 (strongly-disagree) to 4 (strongly-agree) [18]. In addition, we adapted Bray-Hall et al. (2010) confidence measure which assesses students’ confidence in their ability to perform successful care transition practices in the community following discharge (3-items) [19] and items that assess students’ confidence in communicating with interdisciplinary staff, patients from diverse cultural backgrounds and patients whose native language is different than the students (3 items) (5-point scale − 1 = not at all confident; 5 = very confident). Moreover, we asked students to evaluate their knowledge level using a 5-point Likert scale ranging from 1 (I don’t know) to 5 (very much) regarding: common chronic diseases and cultural beliefs of the Galilee communities.

#### Qualitative data

Weekly structured student reports and monthly calls with clinic directors and municipal partners were collected. Additionally, we included an open-ended question in the pre-survey, asking students to indicate “In what areas do you think the program will contribute to your training as a physician”. In the post-survey we added “In what areas do you think the program contributed to your training as a physician”.

To understand the contribution of the program we also conducted post-program telephone interviews with clinic directors and the municipal partners. Clinic directors were also asked to complete a short-structured report on what students did and the main barriers students faced, would they be interested in employing medical students as physician assistant, and their satisfaction with the students’ contribution (ranked between 1-not satisfied to 5-very satisfied).

### Data Analysis

#### Quantitative analysis

Medical students’ responses were analyzed using descriptive statistics. Overall scales were assessed by combining all items of the questionnaire to obtain overall summary assessment scores (alpha-Cronbach > 0.7). “Attitudes assessment” was not assessed as an overall scale due to the small number of items (< 3) and low internal validity (alpha-Cronbach < 0.7). The Wilcoxon Signed Rank test for related samples was used to determine mean differences in pre-and post-survey following completion of the program. McNemar-Bowker Tests were conducted to verify the differences in pre-and post-survey categorical variable proportions, for example, future willingness to integrate into primary care. SPSS statistical software version 27.0 was utilized for this analysis.

#### Qualitative analysis

We performed qualitative thematic analysis of the students’ weekly reports to better understand what they did during their time in clinics or community as well as the open-ended questions in the pre-post surveys regarding the program’s contribution. Thematic analysis was also conducted for the telephone interviews with clinic directors and the municipal partners. The interview included questions such as: “Describe the main tasks that the students performed while working in your clinic?”; “What were the main challenges the students faced?”; “What was the students main contribution to the clinic’s activity?”; “In the future, would you consider hiring a student as a physician’s assistant and if so, for what tasks”. We analyzed the responses of the study participants, categorizing them based on recurring themes and patterns that emerged from the data. Additionally, we selected relevant quotes to illustrate these themes [20].

## Results

### Quantitative results

#### Sample characteristics of medical students

Ninety-one students participated in the program. Eleven (12.10%) pre-clinical students participated in the home visitation program, completing over 840 home visits in 7 Arab villages and towns and distributing over 6,000 community kits. Eighty (87.90%) students in their clinical years were placed in 40 clinics across the region, contributing over 10,600 h. Of these 80 students, 73 matched pairs of surveys were identified, yielding a response rate of 91.25%. The mean age was 29 years, 52% were females, 35% studied in the Faculty’s 3-years study program which that enables students who completed their pre-clinical training abroad to complete their clinical training in Israel, and 52% were students studying in the 4-year study program which accepts students who had completed a first degree. About 64% of those students were in their first clinical year (*n* = 47), while 36% were in their second clinical year (*n* = 26). Table 1 shows the ethnic and religious affiliation of participating students.

**Table 1:** Sample characteristics of medical students from the clinical years at the northern periphery of Israel (*N* = 73)

	N (%)
**Age (Mean; SD)**	28.9 (3.7)
**Gender**	
Male	35 (47.9)
Female	38 (52.1)
**Ethnicity**	
Jews	59 (80.8)
Muslim Arabs	9 (12.3)
Christians Arabs	5 (6.8)
**Study program**	
3-years	35 (47.9)
4-years	38 (52.1)
**Study year**	
First year clinical studies	47 (64.4)
Second year clinical studies	26 (35.6)

SD standard deviation

#### Program contribution to the medical students

The Wilcoxon Signed Rank tests (Table 2) revealed significant differences (*P* < 0.05) regarding knowledge and awareness of working in the community for students post program. For example, students’ knowledge regarding “non-financial barriers to health care” significantly increased from 2.63 to 3.06. Students’ knowledge about “Basic self-care practices as a part of health lifestyles” increased from 2.56 to 2.93 significantly. Post-assessment of students’ knowledge about common chronic disease were also found to be statistically different (*P* < 0.05). For instance, knowledge about diabetes, hyperlipidemia, and hypertension significantly improved from 4.01 to 4.36; 3.80 to 4.15 and 3.96 to 4.23, respectively. In addition, there were significant differences in all knowledge levels (total scale) regarding the cultural beliefs of the Galilee communities. Specifically, students’ knowledge on Muslim-Arabs and Jewish-Orthodox cultural beliefs increased significantly from 3.06 to 3.34 and 3.36 to 3.56, respectively. Confidence Items were rated high by students both pre and post program. However, we found a significant improvement in students’ confidence to communicate properly with multi-professional clinic staff (*P* = 0.042).

**Table 2:** Attitudes, knowledge, and awareness of working in the community for medical students pre and post program

Items	Pre-survey mean (SD)	Post-survey mean (SD)	Z (2-tailed)	P
**Attitude: to what extent do you agree or disagree with each statement:**				
Respect for values that differ from one’s own is necessary to provide competent care	3.71 (0.54)	3.86 (0.38)	-1.95	0.052
HCPs should involve patients and families in the decision-making process	3.59 (0.53)	3.66 (0.51)	-0.96	0.336
**Intention: to what extent do you intend to use this knowledge/skill**				
Working with clients on modification of risk health behaviors	3.18 (0.73)	3.25 (0.62)	-0.83	0.41
Using a community’s demographic statistics to identify key issues	3.25 (0.83)	3.41 (0.70)	-1.59	0.11
Coordination of health care services as a member of multidisciplinary teams	3.66 (0.58)	3.71 (0.51)	-0.67	0.50
Promoting healthy lifestyles in communities	3.44 (0.75)	3.46 (0.66)	-0.46	0.64
*Intention (Total scale)*	3.38 (0.56)	3.44 (0.46)	-0.81	0.42
**How much knowledge and awareness do you have about**:				
Non-financial barriers to health care	2.63 (0.72)	3.06 (0.67)	-3.83	*P* < 0.001
Disparities in health status between periphery and center and between general and minority populations	3.10 (0.75)	3.34 (0.71)	-2.58	0.010*
Sources of support and resources in the community	2.45 (0.71)	2.71 (0.79)	-2.37	0.018*
Basic self-care practices as a part of healthy lifestyles	2.56 (0.78)	2.93 (0.69)	-3.12	0.002*
Chronic disease risk factors	3.41 (0.66)	3.60 (0.57)	-2.06	0.039*
*Knowledge and awareness about community working (Total scale)*	2.83 (0.51)	3.13 (0.50)	-4.12	*P* < 0.001
**Knowledge level of common chronic diseases**				
Diabetes	4.01 (0.84)	4.36 (0.67)	-3.29	0.001*
Hypertension	3.96 (0.86)	4.23 (0.77)	-2.81	0.005*
Hyperlipidemia	3.80 (0.94)	4.15 (0.74)	-3.37	0.001*
Heart disease	3.92 (0.92)	4.25 (0.78)	-3.01	0.002*
COPD	3.80 (0.91)	4.04 (0.82)	-2.43	0.015*
*Knowledge level of common chronic diseases (Total Scale)*	3.90 (0.78)	4.21 (0.65)	-3.82	*P* < 0.001
**Knowledge level of the cultural beliefs of the Galilee populations**				
Muslim Arabs	3.06 (1.15)	3.34 (1.10)	-3.28	0.001*
Christian Arabs	3.04 (1.14)	3.18 (1.03)	-1.33	0.185
Druze	2.70 (1.06)	2.88 (0.96)	-1.62	0.105
Jewish orthodox	3.36 (0.89)	3.56 (0.96)	-2.32	0.020*
Ethiopians	2.30 (0.94)	2.40 (0.98)	-0.93	0.351
*Knowledge level of the cultural beliefs (Total Scale)*	2.89 (0.72)	3.07 (0.74)	-2.64	0.008*
**Familiarity with the attitudes and beliefs about emotional disorders of the Galilee communities**				
Muslim Arabs	2.38 (1.17)	2.88 (1.20)	-3.85	*P* < 0.001
Christian Arabs	2.19 (1.11)	2.78 (1.13)	-4.07	*P* < 0.001
Druze	1.96 (0.90)	2.52 (0.97)	-3.92	*P* < 0.001
Jewish orthodox	2.48 (1.15)	2.93 (1.17)	-3.20	0.001*
Ethiopians	1.96 (0.96)	2.27 (0.85)	-2.43	0.015*
*Familiarity with the attitudes and beliefs (Total Scale)*	2.19 (0.85)	2.68 (0.84)	-4.24	*P* < 0.001
**Confidence: How confident are** **you in your ability to**:				
Review the medications prescribed to the patient and check for any discrepancies	3.66 (1.13)	3.84 (0.94)	-1.59	0.113
Develop a follow-up plan for an unbalanced chronic patient	3.32 (1.15)	3.48 (0.91)	-1.01	0.314
Perform an assessment of the patient’s functional abilities at home	3.66 (0.87)	3.67 (0.99)	-0.42	0.967
Communicate properly with a multi-professional staff at the clinic	4.38 (0.68)	4.58 (0.67)	-2.04	0.042*
Communicate properly with a patient who comes from a culture different from your own	4.43 (0.72)	4.44 (0.67)	-0.91	0.363
Communicate properly with a patient who does not speak your language	3.52 (1.08)	3.55 (0.96)	-0.101	0.910
*Confidence (Total Scale)*	3.83 (0.64)	3.90 (0.61)	-1.12	0.262

*P < 0.05; P values were determined in the two-sided Wilcoxon signed-rank tests related samples; SD: standard deviation; HCPs: Health care providers

The program significantly increased students’ willingness to practice medicine both in primary care as well as in hospital when they finish their studies (*P* = 0.019). Despite the program, the overall scale for intentions of chosen medical specialization that students reported did not change significantly, even though specialization in family medicine increased from 6.8 to 12.3%. Post-program, 60% of students were still undecided as to their preferred geographic work area/region or whether they will stay in the Galilee (Table 3).

**Table 3:** Students career plans pre and post program

	Pre-survey n (%)	Post-survey n (%)	McNemar-Bowker Test	P
**Workplace in the future**				
Hospital	51 (69.9)	38 (52.1)	10.00	0.019*
Community	10 (13.7)	12 (16.4)		
Both	12 (16.4)	23 (31.5)		
**Medical specialization**				
Internal medicine	13 (17.8)	8 (11)	19.67	0.352
Surgery	12 (16.4)	14 (19.2)		
Gynecology	8 (11.0)	10 (13.7)		
Pediatrics	10 (13.7)	9 (12.3)		
Family medicine	5 (6.8)	9 (12.3)		
Other	11 (15.1)	12 (16.4)		
Not yet decided	14 (19.2)	11 (15.1)		
**Work area in Israel**				
North	24 (32.9)	23 (31.5)	2.33	0.675
South	1 (1.4)	2 (2.7)		
Center	7 (9.6)	11 (15.1)		
Not yet decided	41 (56.2)	37 (50.7)		
**Stay in the Galilee region**				
Yes	17 (23.3)	18 (24.7)	0.422	0.810
No	12 (16.4)	14 (19.2)		
Not yet decided	44 (60.3)	41(56.1)		

P-values derived from McNemar-Bowker Tests differences in categorical variable proportions; *P < 0.05

We conducted a sensitivity-analysis to examine the impact of the program on the change in students’ attitudes and knowledge (total scales) comparing students’ clinical year of study (first/second). We found that the program improved first-year clinical students’ “awareness and knowledge of working in the community”; “knowledge of common chronic diseases”; “familiarity with the cultural beliefs and customs of the Galilee communities” and “familiarity with the attitudes and beliefs about emotional disorders of the Galilee communities” significantly (*P* < 0.05). However, among second-year clinical students, the program training significantly improved only their “level of knowledge on common chronic diseases” (*P* = 0.024). In addition, the program increased first clinical year students’ future willingness to integrate into primary care alongside a hospital (*P* = 0.021) as opposed to students in their second-year clinical studies (*P* = 0.20) (data not shown).

### Qualitative results

#### Students’ activities in the clinical and community settings

Thematic analysis of students reports revealed that students gained insights into the unique and important role of primary care, as well as to working with patients and communities of diverse cultural background.

The eleven pre-clinical students worked with disadvantaged municipalities and distributed community kits acknowledged the ongoing challenge communities faced:

*We are currently working in a red city [high number of COVID patients]. People are not wearing masks… they don’t understand the importance of wearing masks when entertaining guests despite all of the information…”.* (Students A, pre-clinical year)

Pre-clinical students working in the community also noted the benefit of having an in-depth understanding and the opportunity to engage with elderly residents of the Arab community:

*The meetings with the Arab elderly community were extremely interesting to me. I felt that this was the biggest value, not only could I engage with them and offer to listen to their problems, but I was really able to understand their hardships.”* (Students B, pre-clinical year).

Clinical students’ remarked on activities in clinics were devoted to telephone calls to patients with chronic diseases to encourage conducting recommended screening routine tests.

*“There were many patients whose treatments were suspended during the Coronavirus, and I tried to provide them with a response. A lot of patients are afraid to go to the clinic to take care of themselves, so they need encouragement not to neglect their condition.”* (Students A, first-clinical year).

Students commented about their worked together with the medical and nursing teams to also monitor chronically ill patients (diabetes, heart-failure, hypertension); performed telephone monitoring of quality indicators (e.g., whether patients preformed mammogram or occult blood tests), and encouraged healthy lifestyle behavior (enrolling in workshops to stop smoking, promoting changes in diet and physical-activity).

*“Together with Dr. B and O, the nurse in charge, we made phone calls to patients whose blood results (diabetes and cholesterol for cardiovascular patients) showed an imbalance; together we tried to understand why they were out of balance, are they careful to take their medications and has anything changed recently perhaps due to the coronavirus period? After the conversations, we thought together about the best follow-up for each* patient.” (Students A, second-clinical year).

Additionally, students’ devoted time to explanations and instructions regarding coronavirus, telephone monitoring of corona patients, and encouraging vaccinations. Other phone calls were devoted to providing emotional support to patients after losing a family member. Students also took part in ongoing clinic activities such as triage, assistance in the absorption of patients, dressings, blood-taking, and medical diagnosis (together with the medical staff).

Students also accompanied medical and nursing clinic teams when conducting home visits to chronic and terminally ill patients (confined patients and home hospitalization) that included: clinical examination, checking for medications discrepancies, and assessing patient’s home environment, independence and functioning in daily life, and their social-emotional support.

### Students’ program summary and contribution

Analysis of student reports after completing indicated that the program’s contribution was in accordance with met and even exceeded students’ expectations. A few students felt frustration for the missed opportunity to meet with patients because their work was conducted mainly by telephone.

*“The program significantly contributed to familiarity with the different types of population in the area and how their culture and mentality affects their health perceptions and cooperation in treatment, and of course their influence regarding the accessibility of the health system for these patients. In addition, I studied and familiarized myself with work processes in community clinics and various services that community medicine provides. I was privileged to work in a multidisciplinary team that includes nursing, medical and administrative staff. Following exposure to health services in the community that I received as part of the program, and especially because I am preparing for a surgical internship that is mostly carried out in a hospital, I believe that I have acquired a lot of important knowledge that I probably would not have been exposed to for many years. I believe this knowledge will help me become a more conscientious physician and work in cooperation with community medicine in the future. Thank you so much for this opportunity!”* (Student B, second-clinical year)”.

Several students noted the program’s specific contribution to their decision-making process regarding future specialization. They noted that the in-depth familiarity with family medicine was valuable, especially given that their clinical training is mainly conducted in the hospital.)

*““I have learned that medicine in the community is very important and even critical. I realized that patient health is well maintained with a detailed and accessible explanation from medical teams.”* (Student B, first-clinical year)

*Family Medicine was not a residency I was considering but in the past few months working in this program in the clinic caused me to see the beauty of this profession and consider this path.”* (Student C, second-clinical year).

### Clinic directors and Community Partners perception of the program

Twenty-nine clinic directors out of forty participating clinics responded. All directors reported high satisfaction (90%) with students. They expressed that the program exposed students to clinical and social aspects, emphasizing the importance of preventive medicine in the community. Clinic directors also report that students became an integral part of clinics’ teams, and they had optimism for recruiting some of the students as future physicians.

*This is a very important project and contributes to exposing the students and deepening their knowledge and understanding of the work of primary care. I believe we will be able to recruit students at the end of their study to family medicine, something that will contribute immensely to improving the health of the community in the future.” (Clinic A, Director)*.

Clinic directors also noted communication and language barriers as a primary challenge, encompassing language differences, communication difficulties and, the need to build trust and rapport with patients from diverse backgrounds.

Dealing with a different culture, different communication, and language problems. Building a relationship with the patients, getting to know the field including a home visit, creating trust and building a two-way relationship with the patients.” (Clinic B, Director).

Time constraints, especially for managing patients with chronic conditions and coordinating appointments, presented significant hurdles within clinic and student schedules. Additionally, addressing unique patient groups, like elderly individuals who avoid primary care visits, demanded collaborative efforts among medical professionals. Furthermore, convincing patients to undergo follow-up tests, perform necessary procedures, and adhere to preventive medicine recommendations posed a substantial challenge. Balancing academics with clinical responsibilities, adapting to a changing healthcare landscape —particularly during the COVID-19 pandemic—engaging in experiential learning through observation, and ensuring patient engagement all contribute to the multifaceted challenges faced by medical students.

Overall, we found that clinic directors recognized the importance of students participating in the clinic’s efforts and were keen to include them as physician assistants should the position become available. It is important to note that supporting physicians through a physician assistant position has been an ongoing discussion in Israel’s health care system.

Our municipal partners also noted a significant impact and notable change in residents’ behavior, especially elderly residents.

## Discussion

Our study shows that the unique opportunity to expose medical students to the community for four months during COVID significantly increased students’ future willingness to practice in the community alongside the hospital. It had a positive significant impact on students’ knowledge regarding common chronic diseases in the community, as well their knowledge of the cultural norms of the communities they worked with.

Community-engaged learning creates opportunities for first-hand and lived experiences with diverse populations and community partners who provide front-line care and program delivery [3, 4, 12]. Medical schools, reported in the pre-COVID, era that these such experiences were key to their students’ deeper understanding of the social intricacies of health disparities [21–23]. Students become much more sensitive to the communities they engage with while learning about the specific circumstances making these populations so vulnerable [24]. Introducing students to the community early in their medical education creates opportunities for them to see and practice interactions with marginalized communities [2]. This practice may help not only cultivate humanistic skills necessary to effectively care for all populations but also to integrate an appreciation for social determinants of health in future clinical years [25–27]. Moreover, based on the literature and our findings, it appears that students experience of being part of a healthcare team does not merely expose them to the primary care setting; but rather it also provides them with an opportunity to gain insight into the unique and important role of primary care in diverse communities [28]. In the context of chronic diseases, health education programs led by medical students within the community can serve as valuable interactive learning tools in medical education while delivering quality primary care for patients with chronic diseases [29].

This bio-psycho-social program generated not only experiences for students but also important insights for a Faculty of medicine deliberately established in a social-geographic peripheral location for purposes of reducing health disparities [13]. While the program enabled students to better understand care for underserved populations, it did not significantly change their attitudes towards staying, living, or practicing medicine in the periphery.

Universities established in the periphery are often considered an engine for economic development [30]. Their major role in regional economic development is in creating a highly skilled workforce. Yet, graduates often don’t stay in the area and move to urban centers, with major reasons being shortage of local employment opportunities and lower compensation [31]. Social attachment to a place, such as proximity to hometowns has been found to be the strongest influence for graduates to stay in the region [32].

Another interesting and important finding from our research was the change in students’ attitudes towards practicing in the community and to family medicine. Northern Ontario’s School of Medicine (NOSM), for example, aims to be socially accountable and has therefore created a curriculum in which community engagement is central. As a result, NOSM has a high rate (61%) of MD graduates who choose residency in family medicine [33]. Studies have shown that having family medicine role models early in medical school as well as participating in rural family medicine influence students’ decision to specialize in family medicine [34]. During our study, the clinical exposure to positive role models may have impacted students to pursue a specialty that they had not previously considered [12].

The present study has several limitations. Students chose to enroll in the program and received a stipend for their participation. This may have led to a selection bias of medical students who were more inclined towards working in the community. Additionally, COVID-19 was viewed as an emergency and, therefore may have created an untypical willingness by students towards working in vulnerable communities. Furthermore, the program served for the faculty as a basis for continued development of experiential learning platforms in which students are eager to participate. The impact on career choice opposed to career interest would need to be measured at the end of medical school or even in later years. In the future, we recommend conducting longitudinal research on this topic. Last, our use of a pre-post design has limitations in the statistical effects measured. However, using triangulated qualitative and quantitative data gives rich insights into our data and findings. Future evaluations that include controls should adjust for various factors, including learning from pre-tests and other life experiences.

To conclude, the unique psycho-social-health program we developed is relevant not only for current times of crisis but builds upon and further fortifies a faculty of medicine’s social accountability to the region which it serves, assisting in improving the quality-of-care, medical students’ knowledge and attitudes towards family medicine, and training future physicians to address those most disadvantaged.

## Data Availability

The datasets generated and/or analysed during the current study are not publicly available to maintain student confidentiality but are available from the corresponding author on reasonable request.
